# A Novel Variant in the *PAH* Gene Causing Phenylketonuria in an Iranian Pedigree

**Published:** 2017

**Authors:** Elaheh Alavinejad, Seyede Zahra Sajedi, Masoumeh Razipour, Mona Entezam, Neda Mohajer, Aria Setoodeh, Saeed Talebi, Mohammad Keramatipour

**Affiliations:** 1. Department of Medical Genetics, Faculty of Medicine, Tehran University of Medical Sciences, Tehran, Iran; 2. Immunology Research Center, Tabriz University of Medical Sciences, Tabriz, Iran; 3. Department of Medical Genetics, Faculty of Medicine, Tabriz University of Medical Sciences, Tabriz, Iran; 4. Department of Pediatrics, Faculty of Medicine, Tehran University of Medical Sciences, Tehran, Iran

**Keywords:** Mutation, Phenylalanine hydroxylase, Phenylketonurias, Population

## Abstract

**Background:**

*Phenylalanine hydroxylase (PAH)* gene is the well-known causative gene for classic Phenylketonuria (PKU) (OMIM#261600) disease, with more than 500 reported mutations. Through this study, a novel mutation in the *PAH* gene in an Iranian pedigree with phenylketonuria was introduced.

**Methods:**

A consanguineous family with a 10-year old affected girl was referred for genetic analysis. Mutation screening of all exons and exon-intron boundaries was performed by Sanger sequencing, and mini haplotype analysis was carried out by genotyping of Short Tandem Repeat (STR) and Variable Number Tandem Repeat (VNTR) alleles.

**Results:**

Mutation analysis revealed a novel homozygous insertion of a single adenine nucleotide at position 335 in exon 3 of the *PAH* gene. Based on the American College of Medical Genetics and Genomics (ACMG) guidelines, the change is interpreted as a pathogenic mutation which produces a premature termination signal (TAA) at codon 113 according to in silico assessments. The mini haplotype analysis showed that this mutation was linked to STR (15) –VNTR (3).

**Conclusion:**

In this study, a novel mutation was reported in a patient who had PKU symptoms without any previously reported mutations in the *PAH* gene (NM_000277.1:p.Asp112Glufs*2) that can be responsible for the classical PKU phenotype in the Iranian population. Detection of novel mutations indicates notable allelic heterogeneity of the PAH locus among this population.

## Introduction

**Figure 1.**
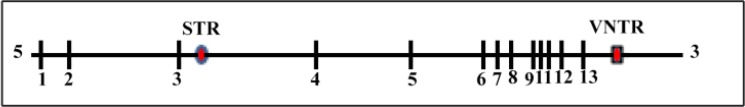
The map of the *PAH* locus indicating exons and two other commonly studied polymorphic markers. The STR in intron 3 and the VNTR in downstream of the last exon are shown.

The *Phenylalanine Hydroxylase (PAH)* gene, being 90–100 *kb*, is embedded in the genomic location 102, 836, 885–102, 958, 410, on the chromosomal region 12q23.2 ^1^. There are several polymorphic markers in the *PAH* gene including a Variable Number Tandem Repeat (VNTR), and an intragenic Short Tandem Repeat (STR), which are located 3 *kb* downstream of the exon 13, and around 200 *bp* downstream of the exon 3, respectively, in addition to eight Restriction Fragment Length Polymorphisms (RFLP) (Figure 1). These markers have provided the possibility of making different haplotypes. What’s more, the combination of VNTR and STR alleles could be used to characterize mini haplotypes at the *PAH* gene ^2^. The genomic sequence and all related data regarding the *PAH* gene are available online at PAHdb ^3^.

Dysfunction or the complete loss of function in the PAH enzyme would cause disturbances in the metabolism of the essential amino acid, phenylalanine, which would in turn cause the Phenylketonuria (PKU) disease ^4^. In addition, the PAH enzyme is responsible for the catabolism of much of the phenylalanine received through diet, the product of which is tyrosine ^5^.

Since mental retardation is the most detrimental outcome of the disease and ought to be prevented by early diagnosis and treatment before the age of 20 days old, screening programs are the most beneficial tools in ensuring the early detection of the patients and have been around since 70 *s* in developed countries and then in developing countries. Thus, with the availability of PKU samples, comprehensive studies have been conducted on the *PAH* gene and its pathogenic allele ^6,7^. Such studies have demonstrated that PAH locus is unusually prone to mutations and more than 500 mutations have been recorded by PAH Mutation Analysis Consortium thus far ^5^.

Despite the high number of pathogenic alleles reported for the PAH locus, there are only less than 10 mutations responsible for certain populations ^1^. In the present study, performing a genetic diagnosis in a consanguineous family with one affected child resulted in introducing a novel mutation in the *PAH* gene.

## Materials and Methods

### Patient and DNA samples

A first cousin consanguineous family with Mazani ethnicity was referred for genetic testing. Their second child was a 10-year old girl who was diagnosed with PKU at the age of 15 months, with the onset of symptoms. The first child of the family was healthy; besides, no PKU had been reported among their relatives. Unfortunately, despite treatment with a restricted diet, the patient’s mental functioning was delayed and she is currently studying in a special school. The patient’s level of phenylalanine at the time of diagnosis was 17 *mg/dl* and now through a restricted diet has plunged to 8 *mg/dl*. Genetic counseling was performed for the family as well.

To extract DNA, blood samples from the whole family was collected after informing them about the process of the project and obtaining informed consent from the parents. The genomic DNA was extracted using the GenEX^TM^genomic Sx kit (GENEALL BIOTECHNOLOGY CO, LTD, South Korea) according to the manufacturer′s instructions. The quality and quantity of DNA were assessed by NanoDrop spectrophotometer (ND-1000) as well as agarose gel electrophoresis.

### PCR amplification and sequencing

New primer sets were designed for amplification of all 13 coding exons of the *PAH* gene and its surrounding sequences with conditions including initial denaturation at 95*°C* for 5 *min* followed by 30 cycles of denaturation at 95*°C* for 30 *s*, annealing temperature at 58–62*°C* (depending on the Tm of the primer sets) for 30 *s*, extension at 72*°C* for 40 *min*, and the final elongation at 72*°C* for 7 *min*. Determination procedure of the sizes of sample bands was performed using 1.5% agarose gel. PCR products were purified using the GeneAllExpinTMkit to improve the quality of sequencing. One-way Sanger Sequencing was carried out using the forward primer for all of the 13 exons of the *PAH* gene and their flanking introns in the ABI 3130 automated sequencer Applied Biosystems (Macrogen Company of Korea). Primers were designed on average 100 base pairs upstream and downstream of exons and PCR products had an average length of 750 *bp*.

Segregation analysis was performed by sequencing the DNA samples of both parents to ensure precision of the study. Besides, for mini haplotype analysis, the regions of VNTR and STR were amplified with labeled primers which were obtained from Goltsov *et al*’s studies ^8,9^. The size of all the PCR products was determined by capillary electrophoresis (provided by the Pishgam Biotech Company, Tehran, Iran (www.Pishgambc.com).

### Variant analysis

The quality of sequencing was then checked by assessing the obtained chromatogram by the Chromas software (version 2.4.1). The pathogenic potential of observed single nucleotide variants was then evaluated using Sift, Provean ^10^, and polyphen2 ^11^ software programs. Indel variations were evaluated by mutation Taster and Combined Annotation Dependent Depletion (CADD) which was introduced as a comprehensive tool in 2014 ^12,13^. New variant was classified according to the latest ACMG guideline ^14^.

## Results

**Figure 2.**
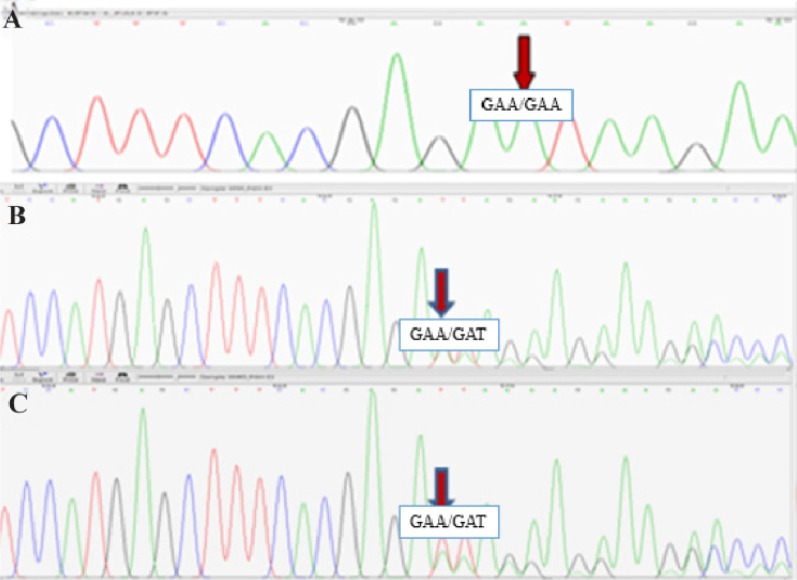
The Sanger Sequencing results. A) The electropherogram of the patient sample shows one adenine nucleotide insertion in a homozygous manner. B, C) The electropherogram of the father and mother samples, respectively. The demonstrated disarrangement results from one adenine nucleotide insertion in a heterozygous manner.

**Figure 3.**
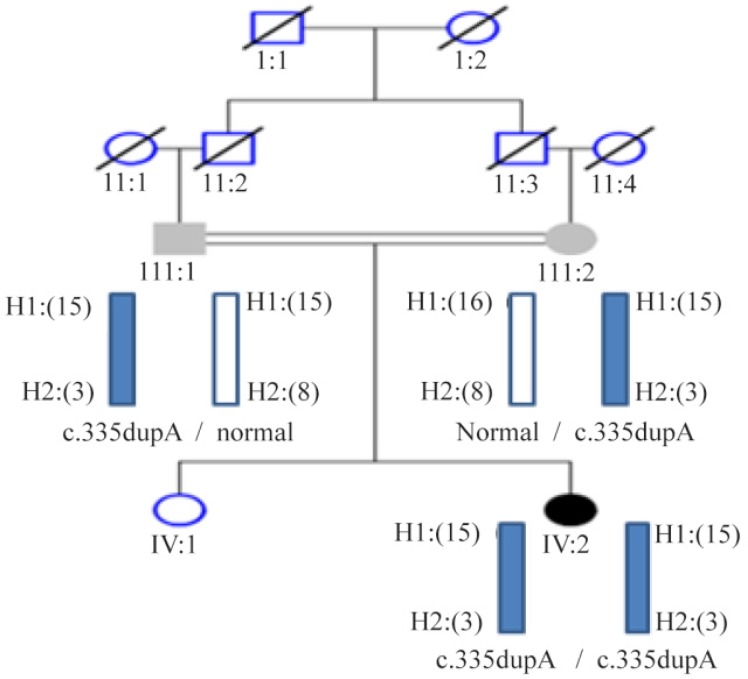
Segregation analysis of the novel mutation and mini haplotypes at the PAH locus in the family. H1: short tandem repeat (STR), H2: variable number tandem repeat (VNTR), numbers in the parenthesis indicate the number of repeats.

Each of the 13 exons of the *PAH* gene and its surrounding sequences, an average of 700 *bp*, were sequenced after amplification and purification. After sequence analysis, all exons except exon 3 did not show any pathogenic variations while the electropherogram analysis of exon 3 indicated the insertion of one adenine nucleotide between codons 335–336 in a homozygous manner (Figure 2A). Moreover, sequence analysis demonstrated the heterozygous state of both parents for the duplication of nucleotide (A) in the same location of the exon 3. The parent’s results were consistent with two overlapping sequences, which consequently confirmed the observed alteration in the patient (Figures 2B and 2C). As for the analysis of mini haplotypes, mutant allele was linked to STR: 15-VNTR: 3 haplotype (Figure 3).

Nomenclature of the variant was performed using Mutalyzer 2.0.11 ( https://mutalyzer.nl/name-checker ). Mutation taster was obtained from the internet to evaluate the NM_000277.1:c.335dup A, p.(Asp112Glufs *2). The result of this analysis could predict that this mutation was disease causing. Also, CADD-score predicted the alteration as deleterious with high probability. According to ACMG classification guidelines, this variant can be predicted as pathogenic. The other variations detected in the proband were also investigated. They were classified as a polymorphism and in this way only the new variant was mutation (Table 1).

**Table 1.** Polymorphisms identified in this study

**Location**	**Nucleotide change**	**dbSNP**	**East Asian frequency**
**Exon-4**	c.353-22C>T	rs2037639	70%
**Exon 5**	c.509+ 101A>C	rs10860933	76%
**Exon 7**	PAH_c.735G>A	rs1042503	76%
**Exon 10**	c.970-195G>A	rs12580432	77%
**Exon 10**	c.1065+97G>A	rs1072528	23%

## Discussion

This study presented a novel finding in a 10-year-old Iranian patient with PKU phenotype. It is a novel mutation without any previous genetic report. By sequence analysis of all the *PAH* gene exons and its surrounding sequences in this family, it showed a duplication (insertion) of one adenine nucleotide in the exon 3 at position 335–336 of the cDNA, for homozygous in the patient, and for heterozygous in her parents. To the best of our knowledge, this mutation has not been reported in any mutation databases to date.

The Iranian population is made up of different races ^2^. Until now, studies carried out on different ethnicities in Iran showed different allelic frequencies. Besides, novel mutations are reported in the most of these studies. As an example, a study carried out in 2014 within the Kurd race in the city of Kermanshah introduced 2 novel polymorphisms and 2 novel mutations ^15^. The above mentioned study as well as our study suggests that the PAH alleles are considerably heterogeneous among the Iranian population and there are some mutations which are quite specific to this population. Thus, it can be predicted that more novel mutations may occur in Iranian population which have still gone undetected. Hence, further investigation is needed to shed light on the issue.

Among the heterogeneous PAH alleles, missense mutations are the most common pathologic forms whereas small deletions, splice region variants, nonsense mutations, small insertions and large deletions occur much less frequently ^16^. In our study, the mutation type was insertion/duplication in which one base pair duplication leads to alteration of aspartic acid to glutamic acid in codon 112 and subsequently frameshift mutation occurs which introduces a Premature Termination Codon (PTC). This intern is predicted to cause nonsense-mediated decay (NMD), which can suggest the lack of any functional protein.

**Figure 4.**
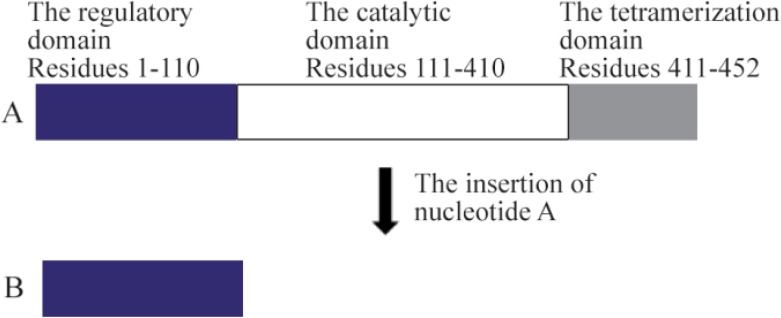
The PAH protein structure. A) The normal structure. B) The truncated structure due to c.335dupA mutation: the premature stop codon generated by the adenine duplication eliminates the catalytic and the tetramerization domain (a region about 340 amino acids).

PAH enzyme (PheOH, Phenylalanine 4-monooxy-genase.EC 1.14.16.1) consists of three domains: the regulatory domain (residues 1–110), the catalytic domain (residues 111–410), and the tetramerization domain (residues 411–452) ^17,18^. In our study, the premature stop codon results in the elimination of catalytic and tetramerization domains which is conducive to the activation of NMD mechanism in the nucleus and this would eliminate mRNA and consequently impairs the function of the *PAH* gene and makes it a null allele ^19,20^ (Figure 4). Therefore, using computational predictive data, population data, as well as segregation analysis of NM_000277.1:c.335dupA, this mutation is interpreted as pathogenic and can subsequently create the PKU phenotype. Nevertheless, it is noteworthy to recommend *in vitro* expression study, for the final approval of the pathogenic effects of this novel mutation.

## Conclusion

In conclusion, a novel pathogenic mutation was presented in an Iranian patient with PKU symptoms. This is a frameshift mutation, c.335dupA, which, very likely destroyed *PAH* gene function completely. Furthermore, this finding could extend the mutation spectrum of *PAH* gene in Iranian population and provide better insight into genetic counseling of Phenylketonuria disease.
